# Assessing Irritability in Primary School-Aged Children: How to Test and Whom to Ask

**DOI:** 10.3390/children12121583

**Published:** 2025-11-21

**Authors:** Susann Tayaranian Djeyhuni, Alexander Prehn-Kristensen, Kamila Jauch-Chara, Manuel Munz

**Affiliations:** 1 Department of Child and Adolescent Psychiatry, Center for Integrative Psychiatry, School of Medicine, 24105 Kiel, Germany; susann.tayaranian@uksh.de (S.T.D.); alexander.prehn-kristensen@uksh.de (A.P.-K.); 2 Department of Psychology, MSH Medical School Hamburg, University of Applied Sciences and Medical University, 20457 Hamburg, Germany; 3 Vincera Klinik Bad Waldsee GmbH, Private Clinic for Psychosomatic Medicine und Psychotherapy, 88339 Bad Waldsee, Germany; k.jauch-chara@vincera-kliniken.de; 4 Clinic for Child and Adolescent Psychiatry, Psychotherapy and Psychosomatics, Center for Integrative Psychiatry, School of Medicine, 24105 Kiel, Germany

**Keywords:** irritability, frustration, low-threshold assessment, school environment, universal screening, targeted prevention

## Abstract

**Highlights:**

**What are the main findings?**

• In primary school-aged children, irritability can reliably be measured with a German Version of a frustration Go/-No-Go task.
• Teacher ratings demonstrated the highest predictive validity for irritability among all informants.

**What is the implication of the main finding?**

• Irritability is a good indication of commencing mental problems or diseases.
• Its easy, quick and reliable measurement enables targeted prevention for children at risk.

**Abstract:**

**Background/Objectives**: An increasing number of children and adolescents with mental health issues across many countries highlights the need for effective, accessible prevention strategies. Within the Research Domain Criteria framework, irritability—an emotional response to expected but blocked rewards—has been identified as an early indicator of declining mental well-being before the onset of mental disorders. Developing reliable yet resource-efficient methods to assess irritability is an important first step toward targeted prevention. **Methods**: We tested the German Version of a frustration Go/No-Go task enabling to deliberately induce frustration through expected but blocked rewards in N = 68 children aged eight to ten (mean age = 8.9 years, SD = 0.7; 36 females) and parallelly assessed irritability with the 7-item low cost Affective Reactivity Index (ARI) with ratings of parents, teachers and children. **Results**: The German Version of the frustration Go/No-Go task proved reliable and valid. Teacher ratings demonstrated the highest predictive validity for irritability among all informants. **Conclusions**: Both the 7-item ARI, rated by teachers, and the frustration Go/No-Go task are recommended for assessing irritability. In the future, irritability assessments should be implemented for all primary school-aged children. Based on the components that contribute to irritability, targeted prevention measures should be offered.

## 1. Introduction

Numerous international studies [1], including large-scale surveys in Germany [2], have reported an increase in mental health problems among children and adolescents, following the COVID-19 pandemic. While a decline in mental well-being was observed in adolescents during the later stages of primary education [2], preschool-aged children exhibited difficulties with self-regulation, manifested by increased sadness, anger, sleep disturbances, behavioral problems, inattention, as well as somatic complaints such as headaches and stomachaches [3,4]. At the same time, teachers have reported a significant increase in problems with concentration and motivation, greater physical restlessness, and a decline in social skills and self-regulation among primary school children [5,6]. Alongside the rise in subclinical behavioral issues, there has also been an increase in clinical diagnoses and outpatient cases [7], including higher rates of depression and a growing need for psychotherapeutic treatment [8]. The alarming rise in mental health issues among adolescents and even children poses significant challenges both in the educational setting—where school professionals feel overwhelmed in their daily work, as do the students themselves [5]—and in the healthcare system, where current treatment capacities fall far short of demand [9]. From a prognostic perspective, and according to the World Health Organization (WHO), primary prevention may offer effective strategies to reduce the impact and consequences of stress and mental health disorders [10].

In recent years, the construct of irritability, defined as a “proneness to anger” [11], has gained increasing attention in research on the early detection of emerging mental disorders [12]. Representing a dimensional spectrum—from low or normative levels to atypically high severity [13]—studies have shown that elevated irritability in childhood predicts the later development of mental disorders and even an increased risk of suicide in adulthood [14]. Additionally, functional imaging data indicate abnormal activation in several brain networks important for cognitive control and emotion regulation—including the amygdala, striatum, anterior cingulate cortex, and lateral prefrontal cortex—suggesting underlying neural mechanisms of irritability [13]. Therefore, elevated irritability can be considered both a general marker of increased risk for mental disorders and a promising, measurable target for indicated prevention in children exhibiting atypical levels of irritability.

The Research Domain Criteria (RDoC) initiative in personalized psychiatry stands in contrast to the traditional diagnostic classifications of the Diagnostic and Statistical Manual of Mental Disorders (DSM-5) [15], by adopting a translational, biologically informed, and less syndrome-based approach. This innovative approach links genetic, neural, behavioral and environmental mechanisms across the spectrum of normal and abnormal functioning, potentially enhancing precision in identifying underlying processes and improving cross-species research translation. Ultimately, it might support the development of more biologically informed and more precisely targeted interventions [16]. Within the RDoC framework, irritability corresponds to the construct of frustrative non-reward, which is defined as the affective response that occurs when an individual is blocked from obtaining an expected reward [12]. Reflecting the continuum of irritability, there are interindividual differences in responses to laboratory tasks designed to elicit and measure frustrative non-reward [17]. While typically developing children (TDC) exhibit frustration as a normative affective response to blocked goal attainment, children with higher levels of irritability have been found to show increased physiological arousal [18,19], greater self-reported frustration [20,21], and a reduced ability to shift attention [20]. Among various experimental tasks, the Frustration Go/No-Go task—which measures the effects of frustration on cognitive control [22]—has proven to be sufficiently reliable and valid for use with school-aged children, particularly in its English version that includes the child version of the Positive and Negative Affect Schedule (PANAS-C) [23].

While the Strengths and Difficulties Questionnaire (SDQ) [24] is widely used across all continents, with its global score often serving as an indicator of overall psychological well-being, the Affective Reactivity Index (ARI) [25] was specifically developed as a brief, efficient, and easy-to-use scale for measuring irritability. As outlined above, increased affective dysregulation places a significant burden on everyday school life for both children and teaching staff. Given that children spend long hours at school, teachers, though potentially influenced by context-specificity of observations (school environment), may serve as reliable informants when assessing irritability, drawing on their experience with many students over their careers—unlike parents or the children themselves, whose subjective ratings can be influenced by various factors, such as developmental stages. While the parent-report ARI has shown similarly promising psychometric properties across various cultural contexts [26,27,28,29], to the best of our knowledge, the Frustration Go/No-Go task has not yet been tested in any language other than English.

As irritability is a well-established construct within the RDoC framework, with prognostic links to mental well-being in adulthood [14], its efficient and reliable assessment in children holds potential for identifying emerging mental health problems or developing diagnoses. It also provides a measurable target for early preventive interventions. Based on findings from English-speaking countries, irritability can be reliably and validly measured using the Frustration Go/No-Go task [22] as well as a brief 7-item questionnaire completed by parents and children themselves [30]. In the present study, we employed the Frustration Go/No-Go task with instructions translated into German, along with the German version of the PANAS-C [31], in a sample of German-speaking children aged eight to ten years. We also assessed overall mental well-being using the SDQ [24] through self- and parent-reports, as well as irritability using the German version of the ARI [25] via self-, parent-, and teacher-reports. We hypothesized that (1) the Frustration Go/No-Go task can validly and reliably measure the response to frustrative non-reward as an indicator of irritability, and (2) teachers would provide the most accurate assessments of irritability in the sample of children compared to parents or the children themselves.

## 2. Materials and Methods

### 2.1. Procedure

As part of a study employing a quasi-experimental, single-arm repeated measures design to evaluate the feasibility and effectiveness of a school-based intervention program (Stress Arousal Regulation Treatment for Kids) (DRKS00031448, [32])—the results of which are reported elsewhere [33]—pre- and post-intervention neuropsychological data were collected from a sub-sample of pupils at one specific school. This study was conducted within the framework of the “Project for Early Intervention and Prevention of Coronary Mental Illness in Adolescents (PRO-Jung),” funded by the Schleswig-Holstein state government [34]. Socio-demographic data were gathered from parents, children, and teachers, and children’s mental health was assessed using validated questionnaires.

### 2.2. Participants

Data were collected from 68 third-grade children (mean age = 8.9 years, SD = 0.7, range: 8–10 years; 36 females) and 46 parents (mean age = 42.97 years, SD = 5.4, range: 30–53; 38 mothers, 8 fathers). Among the parents, 45 held German citizenship and one mother had Russian citizenship. The distribution of the parents’ highest educational qualifications was as follows: none had no qualification or only a special school leaving certificate; one parent had a lower secondary school leaving certificate; 11 had an intermediate secondary school leaving certificate; 11 held a high school diploma; and 23 parents had a university degree. In addition to the children and parents, four female teachers (mean age = 57.5 years, SD = 3.54, range: 55–60) participated in the study. Their professional experience ranged from 27 to 33 years (mean = 30, SD = 4.24), and they assessed a total of 65 pupils.

### 2.3. Outcome Measures

Sensitivity to blocked goal-attainment was assessed using the previously validated Frustration Go/No-Go paradigm (screen task) developed by Seymour et al. [22]. This specific task was adapted for use in German-speaking populations. This adaptation involved translating the task instructions, the feedback messages following correct and incorrect responses, and the items of the PANAS-C questionnaire. Consistent with the original study [22], frustration elicited within the paradigm was assessed using the German version of the questionnaire by Breyer and Bluemke (PANAS-C) [31]. As part of the adaptation process, the translated task was successfully piloted with a small sample (n = 5). Furthermore, before the first experimental block, each participant completed 12 practice trials to identify and correct any potential misunderstandings of the instructions. The paradigm consists of three blocks: the first block presents a standard Go/No-Go task (GNG) without frustration; the second block presents the same task but explicitly induces frustrative non-reward (i.e., children fail to achieve their goal even though they perform all tasks correctly); and the third block again presents the familiar task without frustration. As in the original study [22], the degree of frustration elicited by the paradigm was assessed using the Positive and Negative Affect Schedule for Children (PANAS-C), the German version of the questionnaire by Breyer and Bluemke [31]. The PANAS-C was administered before the first block (baseline) and after each of the three blocks. Sensitivity to the absence of expected rewards was therefore operationalized as the level of self-reported frustration. The entire task took approximately 25 min to complete.

Irritability was measured using the Affective Reactivity Index (ARI) questionnaire [25], administered both as a self-report for children and as external assessments completed by parents and teachers. Each version consists of seven items rated on a three-point Likert scale (strongly disagree/partially agree/strongly agree). The ARI has been widely used in studies on irritability [22,25] and demonstrates good internal consistency, high validity, and a robust one-factor structure.

The Strengths and Difficulties Questionnaire (SDQ)—German adaptation—measures prosocial behavior and behavioral problems in children and adolescents [34,35]. It can be used for participants aged 4 to 16 years. The SDQ includes 25 items that are allocated to five scales: four problem scales—emotional problems (EP), conduct problems (CP), hyperactivity (Hyp), and peer problems (PP)—and one prosocial behavior scale (Pro). The four problem scales are summed to yield a total difficulties score. Each item can be rated as not true, somewhat true, or certainly true. Each of the five scales produces a raw score ranging from 0 to 10, while the total difficulties score ranges from 0 to 40. Cut-off values for the four subscales and the total difficulties score allow classification into three categories: normal (0–15), borderline (16–19), or abnormal (20–40). These thresholds were determined based on normative data, such that 80% of children are classified as normal, 10% as borderline, and 10% as abnormal [24]. The psychometric properties of the parent-report version used in the present study are considered good.

### 2.4. Data Analysis and Statistics

Statistical analysis was performed using SPSS (IBM SPSS Statistics 29.0.0.0) and Stata (StataCorp LLC, Stata/MP 17.0).

First, we wanted to check whether the transfer of the frustration GNG task from English to German was successful and whether we could replicate the results of the study by Seymour et al. [22] with regard to the errors made under frustration. For this purpose, *t*-tests were calculated for the mean values of the errors made in the three blocks. In addition, a one-way ANOVA was calculated to test the significance of the differences in self-reported frustration at the four measurement points. The subsequent objective was to examine which measured variables most strongly predict general psychological stress in children. This was operationalized within the total value across the 4 problem scales of the SDQ [35]. As the statistical requirements of multiple linear regression were not sufficiently fulfilled, the correlation coefficients were determined. On the one hand, the children’s ARI assessments of themselves, their parents, their teachers and the mean value of all three assessments were used as measured variables. On the other hand, the following measured values from the frustration Go/No-Go task [22] were integrated into the calculations: Mean value of No-Go errors made across all 3 blocks, mean value of reaction times across all three blocks, and mean value of self-rated frustration (PANAS-C) [31] across the three blocks and baseline. Questionnaire data were collected using an online survey tool. In order to be able to generate meaningful results, the survey tool was configured so that all questions had to be answered for completion, ensuring that no missing data were present in the completed responses. Surveys that were terminated prematurely and therefore incomplete were excluded from the analyses. Consequently, ARI [25] assessments from the children themselves, the parents and the teachers, the SDQ data from the parents and the neuropsychological data from the Go/No-Go task. In order to capture a possible gender effect, in addition to correlations across the entire sample, correlations were also calculated separately for the females and the males.

## 3. Results

**Figure 1:**
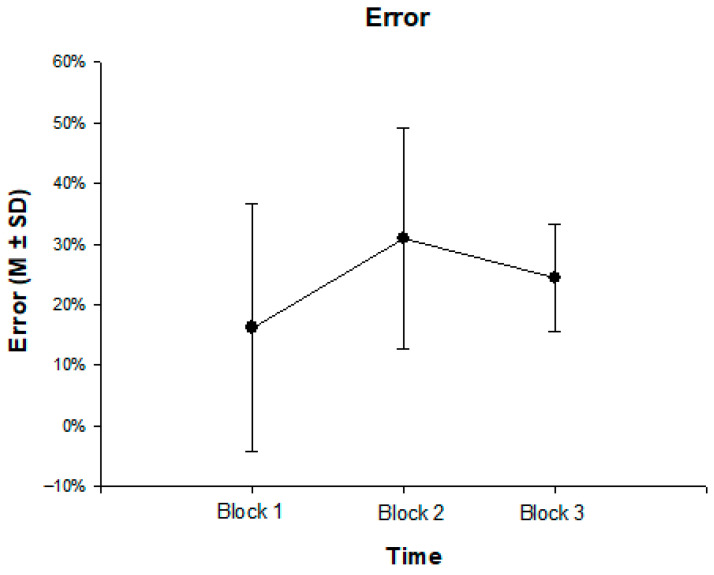
Error Percentage in the No-Go tasks across all three task blocks in the frustration Go/No-Go task (N = 68, block 1: M = 16%, SD = 20.5%; block 2: M = 30.9%, SD = 18.2%; block 3: M = 24.5%, SD = 8.9%).

Regarding deliberate frustration, differences in Go/No-Go error rates were calculated using a repeated measure ANOVA (rmANOVA) with the within factor “block”, which yielded a main effect of block [F(2, 62) = 32.09, *p* < 0.001, η^2^ = 0.32, Power = 0.99]. The factor levels accounted for 32.4% of the variance. As shown in Figure 1, the mean error rate increased from 16.0% (SD = 20.5%) in the first block to 30.9% (SD = 18.2%) in the frustration block and then decreased to 24.5% (SD = 8.9%) in the final block. The rmANOVA was decomposed with post hoc pairwise *t*-tests (block 1–block 2: mean difference = −14.9%, 95%-CI [−194%, −11%], *p* < 0.001; block 1–block 3: mean difference = 8.5%, 95%-CI [−14%, −3%], *p* < 0.001; block 2–block 3: mean difference = 6.4%, 95%-CI [2%, 12%], *p* < 0.001).

**Figure 2:**
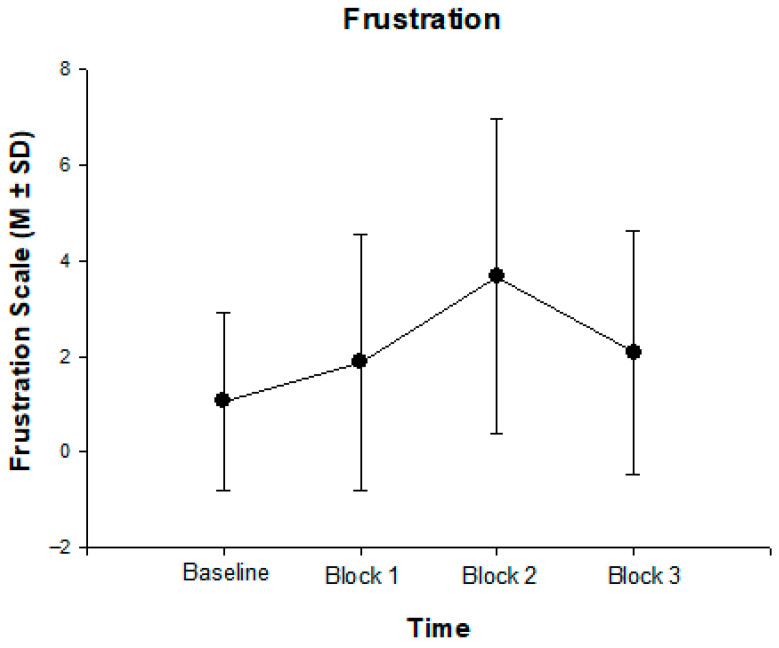
Changes in self-reported frustration across the four measurement points of the Go/No-Go task. (N = 68, baseline: M = 1.07, SD = 1.86; block 1: M = 1.90, SD = 2.67; block 2: M = 2.10, SD = 2.56; block 3: M = 2.10, SD = 2.56).

Regarding self-reported frustration from baseline to block 3, rmANOVA with the within factor “block” revealed a significant effect of block on frustration ratings, [F(3, 61) = 20.32, *p* < 0.001, η^2^ = 0.23, Power = 0.99], indicating that 23% of the variance in frustration was explained by measurement time. As shown in Figure 2, participants’ mean frustration ratings at baseline averaged 1.07 (SD = 1.86) and increased to 1.90 (SD = 2.67) after block 1. During the frustration condition (block 2), the mean frustration level rose sharply to 3.69 (SD = 3.29) and subsequently decreased to 2.10 (SD = 2.56) at the final measurement (block 3). The rmANOVA was decomposed with post hoc pairwise *t*-tests (baseline–block 2: mean difference = 2.60, 95%-CI [1.49, 3.71], *p* < 0.001; baseline–block 3: mean difference = 1.02, 95%-CI [0.12, 1.97], *p* < 0.001; block 1–block 2: mean difference = 1.79, 95%-CI [0.75, 2.84], *p* < 0.001; block 1–block 2: mean difference = 1.79, 95%-CI [0.75, 2.84], *p* < 0.001; block 2–block 3: mean difference = 1.59, 95%-CI [0.65, 2.56], *p* < 0.001). As expected, the frustration Go/No-Go task elicited a marked increase in frustration during the frustration block. Although frustration levels declined thereafter, they did not return to baseline, suggesting that the pattern observed in the original study by Seymour et al. [22] was successfully replicated in this German-language sample.

The results of the Spearman correlations of the joint data of females and males can be found in Table 1.

As shown in Table 1, teacher ratings on the ARI were significantly correlated with the SDQ (*p* < 0.001), whereas parent ratings (*p* > 0.05) and child self-reports did not show significant associations with the SDQ. All three ARI informant scores were, however, strongly correlated with the composite mean score derived from them. Moreover, the overall mean ARI score was significantly associated with both the SDQ total score and self-reported frustration in the Go/No-Go task. Notably, only teacher ratings on the ARI were correlated with both No-Go errors and self-reported frustration. Overall, with correlation coefficients ranging from 0.41 to 0.72, these findings indicate effects of moderate magnitude.

For the female subsample, only teacher-rated ARI scores (*p* < 0.05) and the composite ARI mean score (*p* < 0.05) were significantly correlated with the SDQ. Parent- and teacher-rated ARI scores were both significantly associated with the overall ARI mean, whereas the child self-report was not. Additionally, only the teacher-rated ARI showed a significant correlation with No-Go errors in the screen task. Spearman correlation results for the female subsample are presented in Table 2.

In the male subsample, presented in Table 3, the SDQ was significantly correlated with the teacher-rated ARI, the composite ARI mean (*p* < 0.001), and self-reported frustration in the Go/No-Go task (*p* < 0.05). Strong associations were also observed between each individual ARI score and the overall ARI mean. Moreover, self-reported frustration was significantly correlated with both the teacher-rated ARI and the composite ARI mean.

**Table 3:** Spearman correlations of the Affective Reactivity Index (ARI) [25], frustration ratings and No-Go Errors in the frustration Go/No-Go tasks and the German adaptation of the Strengths and Difficulties Questionnaire (SDQ) [31,32] of the data of males.

	ARI Parents	ARI Teachers	ARI Children	ARI (M)	Frustration (M)	No-Go Errors (M)	Reaction Times (M)	SDQ
**ARI Parents**	1							
**ARI Teachers**	0.484	1						
**ARI Children**	−0.739 **	0.676 **	1					
**ARI (M)**	0.830 **	0.821 **	0.926 **	1				
**Frustration (M)**	0.337	0.619 *	0.309	0.534 *	1			
**No-Go Errors (M)**	0.238	0.317	0.272	0.304	0.030	1		
**Reaction Times (M)**	0.303	−0.066	0.002	0.127	0.169	−0.161	1	
**SDQ**	0.519	0.635 *	0.412	0.569 *	0.609 *	0.115	−0.029	1

* *p* < 0.05, ** *p* < 0.001.

## 4. Discussion

In the present study, we demonstrated that the frustration Go/No-Go task developed by Seymour et al. [22] is applicable to a German-speaking, non-clinical sample of primary school–aged children. Frustration was reliably induced, as evidenced by increased error rates in the frustration block and higher self-reported frustration on the German version of the Positive and Negative Affect Schedule for Children (PANAS-C) [31]. Furthermore, aggregate scores on the German version of the Affective Reactivity Index (ARI) [25] were significantly correlated with global scores on the Strengths and Difficulties Questionnaire (SDQ) [24]. Notably, only teacher-rated irritability on the ARI was associated with both the number of errors in the frustration block and self-reported frustration levels.

When results were analyzed separately by gender, boys’ ARI scores were correlated with self-reported frustration in the frustration Go/No-Go task, whereas girls’ ARI scores were associated with error rates in the frustration block. These gender-specific patterns may suggest that boys tend to exhibit a more verbal or affective response to frustration, while girls may manifest frustration behaviorally through performance errors, potentially reflecting heightened arousal [18,19] and impaired attentional shifting [20]. These gender-specific findings warrant confirmation in larger-scale behavioral studies. Moreover, investigations of the underlying neural mechanisms through neuroimaging and neurophysiological approaches may contribute to a more profound biological understanding. Taken together, these findings indicate that gender-related differences should be carefully considered when assessing irritability in children. In boys, irritability assessment may rely predominantly on verbal responses, indicating that therapeutic strategies should emphasize the regulation of affective reactions to blocked rewards. In girls, irritability assessment may depend more strongly on performance errors and arousal-related biases, implying that treatment approaches should focus on attenuating arousal following blocked rewards [18,19]. Meanwhile, both girls and boys may benefit from interventions targeting attentional shifting [20].

Assessing and evaluating irritability as a common pathway leading to psychological distress, mental disorders, and functional impairment is an important step in identifying at-risk populations and developing effective prevention strategies. Primary school–aged children are in a formative developmental stage and may therefore be particularly responsive to interventions that promote mental well-being and resilience [36]. Yet, the question remains: how, and through whom, can irritability be best assessed in this age group?

Our findings suggest that teachers are particularly well suited to evaluate stress-related behaviors that are relevant to children’s daily functioning in school contexts. Unlike parents, who can observe only their own children, teachers are able to compare behavior across multiple children, offering a broader and more normative perspective. Moreover, children at this age often have difficulty evaluating their own behavior in relation to that of their peers [37]. Given that the Affective Reactivity Index (ARI) is a resource-efficient tool for assessing irritability, the use of teacher ratings within school settings represents a pragmatic and effective approach to identifying children who may benefit from early preventive interventions.

Similarly, the frustration Go/No-Go task [22] proved to be a reliable and valid experimental measure of irritability in a German-speaking sample, suggesting that further adaptations to other languages could be feasible. However, due to the organizational and technical demands of the task, its current format may be better suited for controlled laboratory settings than for everyday use. Yet, considering the child-friendly design of the frustration Go/No-Go task with its penguin-and-igloo outfit [22] and the increasing familiarity of children with digital technologies, developing a mobile application based on this task could allow for playful, accessible, and accurate assessment of irritability in real-world contexts.

As discussed above, irritability represents a central transdiagnostic construct and may serve as a key pathway to various forms of later psychopathology [14]. Early identification of children showing subclinical yet elevated irritability should therefore be followed by targeted preventive interventions. Conceptually, irritability—as reflected in heightened arousal [18,19], increased self-reported frustration [20,21], and reduced attentional shifting [20]—can be addressed through training programs that strengthen cognitive control [22]. We propose that such interventions should be developed in a playful, engaging format and regularly evaluated using measures such as the frustration Go/No-Go task or the seven-item ARI teacher rating. Deliberate, targeted prevention strategies offer a resource-efficient, effective, and goal-oriented approach to mitigating the growing burden of mental ill-health in children [1].

Several limitations should be acknowledged. Collecting data from children attending a single primary school within a relatively homogeneous regional context limits the generalizability of our findings. Since reactions to expected but blocked rewards may depend on cultural and social contexts, further studies are needed to assess the universality of the frustration Go/No-Go task. Furthermore, although teachers tend to show high predictive validity for irritability among informants, their ratings might be biased, as they observe the children only during certain parts of the day and in specific situations. Additionally, cultural influences on teachers’ perceptions and interpretations of irritability may also account for differences compared to, for example, parents’ ratings.

## 5. Conclusions

In conclusion, the German version of the frustration Go/No-Go task is a valid tool for assessing irritability, and its practical implementation could potentially be enhanced through digital technologies in the future. From a cost- and resource-efficiency perspective, teacher-rated assessments using the ARI questionnaire offer a practical and easily implementable approach that can be integrated into routine school practices. Our findings suggest that teacher-rated ARI screenings may represent a promising tool for universal, school-based monitoring strategies—for instance, by implementing annual ARI assessments and providing primary preventive interventions when scores exceed age-related normative thresholds. Future research should extend beyond primary school-aged children and include more diverse populations, enabling broader and more generalizable recommendations. Additionally, longitudinal studies examining developmental trajectories of irritability and targeted prevention would provide valuable prospective data.

## Figures and Tables

**Table 1:** Spearman correlations of the Affective Reactivity Index (ARI) [25], frustration ratings and No-Go Errors in the frustration Go/No-Go tasks and the German adaptation of the Strengths and Difficulties Questionnaire (SDQ) [33,34] of the joint data of females and males.

	ARI Parents	ARI Teachers	ARI Children	ARI (M)	Frustration (M)	No-Go Errors (M)	Reaction Times (M)	SDQ
**ARI Parents**	1							
**ARI Teachers**	0.256	1						
**ARI Children**	0.203	0.235	1					
**ARI (M)**	0.715 **	0.670 **	0.616 **	1				
**Frustration (M)**	0.081	0.441 **	0.144	0.264	1			
**No-Go Errors (M)**	0.007	0.411 *	0.208	0.257	0.251	1		
**Reaction Times (M)**	0.155	0.038	−0.202	−0.010	−0.010	−0.221	1	
**SDQ**	0.422 *	0.516 **	0.121	0.495 **	0.406 *	0.228	0.100	1

* *p* < 0.05, ** *p* < 0.001.

**Table 2:** Spearman correlations of the Affective Reactivity Index (ARI) [25], frustration ratings and No-Go Errors in the frustration Go/No-Go tasks and the German adaptation of the Strengths and Difficulties Questionnaire (SDQ) [31,32] of the data of females.

	ARI Parents	ARI Teachers	ARI Children	ARI (M)	Frustration (M)	No-Go Errors (M)	Reaction Times (M)	SDQ
**ARI Parents**	1							
**ARI Teachers**	0.134	1						
**ARI Children**	−0.137	−0.129	1					
**ARI (M)**	0.635 **	0.582 **	0.331	1				
**Frustration (M)**	0.104	0.262	−0.027	0.023	1			
**No-Go Errors (M)**	−0.143	0.469 *	0.095	0.175	0.387	1		
**Reaction Times (M)**	0.090	0.125	−0.403	−0.043	−0.168	−0.259	1	
**SDQ**	0.411	0.463 *	−0.034	0.511 *	0.272	0.273	0.141	1

* *p* < 0.05, ** *p* < 0.001.

## Data Availability

The data presented in this study are worthy of protection and are available on request from the corresponding author.

## Abbreviations

The following abbreviations are used in this manuscript:

ANOVA	Analysis of Variance
WHO	World Health Organisation
GNG	Go/No-Go Task
ARI	Affective Reactivity Index
SDQ	Strengths and Difficulties Questionnaire
PANAS-C	Positive and Negative Affect Schedule
TDC	Typically developing children
RDoC	Research Domain Criteria
